# Effect of Poly (Vinyl Alcohol) on the Properties of Cold-Setting Melamine–Urea–Formaldehyde Resin Adhesive

**DOI:** 10.3390/ma18225125

**Published:** 2025-11-11

**Authors:** Jiankun Liang, Bengang Zhang, Longxu Wu, Yuqi Yang, Caihong Long, Zhixian Song, Hui Yang, Zhigang Wu

**Affiliations:** 1 College of Civil Engineering, Kaili University, Qiandongnan 556011, China; dushimensheng@126.com; 2 Forest Products Industry Research Institute, Jiangxi Academy of Forestry Sciences, Nanchang 330013, China; zhangbengang@jxlky.cn; 3 College of Forestry, Guizhou University, Guiyang 550025, China; wlx18786427724@163.com (L.W.); 15180721265@163.com (Y.Y.); chlong0927@163.com (C.L.);

**Keywords:** melamine-urea-formaldehyde resin, poly (vinyl alcohol), tensile property, bonding strength, water resistance, microscopic morphology

## Abstract

This study investigates how poly (vinyl alcohol) (PVA) influences melamine–urea–formaldehyde (MUF) resin, particularly regarding tensile properties, bonding strength, water resistance, curing temperature, chemical structure, and microscopic morphology. By altering the PVA content, we observed changes in the tensile strength and elongation of MUF resin. The tensile strength peaked at a 2% PVA addition. PVA significantly enhanced the dry, cold water, and boiling water bonding strengths of MUF resin, with the most notable effect at a 10% addition. A low PVA addition (2%) notably improved the water resistance of glued wood. Differential scanning calorimetry revealed that PVA increased the curing temperature of MUF resin, though excessive PVA led to a decrease. Nuclear magnetic resonance analysis showed changes in chemical bonds after PVA modification, indicating increased polymerization. X-ray diffraction and scanning electron microscopy analyses further confirmed the effects of PVA on the crystal structure and microscopic morphology of MUF resin, with modified resins exhibiting higher toughness fracture characteristics. These findings suggest that PVA can effectively enhance the overall performance of MUF resin, making it more suitable for applications of glued wood.

## 1. Introduction

Glued wood, an efficient way to utilize wood, maintains the wood’s physical structure through processes such as drying and defect removal. It shows excellent performance in compression resistance, tensile strength, and material uniformity. Glued wood optimizes wood usage and enhances overall efficiency. It has become widely adopted and developed in technologically advanced countries and is now the mainstream form of wooden structural buildings [1,2,3,4,5,6,7]. Adhesives are crucial in glued wood manufacturing as they directly impact its water resistance, durability, and weather resistance. Therefore, enhancing adhesive properties is essential for producing high-quality glued wood [8,9,10].

Currently, various adhesives are available for glued wood, including resorcinol-based, isocyanate-based, melamine-based, polyvinyl acetate-based, urea–formaldehyde resin, and protein-based adhesives [11,12,13,14,15,16]. Among them, melamine–urea–formaldehyde (MUF) copolymer resin, known for its light color and cost-effectiveness, is the mainstream melamine-based adhesive for glued wood production in North America and Europe. However, after curing, MUF resin has issues like short intramolecular network chains, high cross-linking density, and large spatial steric hindrance, leading to high rigidity, brittleness, and poor impact resistance, posing potential safety hazards in glued wood structural applications [17,18,19,20].

To address MUF resin’s brittleness, researchers have mainly focused on reducing cross-linking density to enhance toughness. Common methods include adding physical barriers like cellulose powder, starch, polyvinyl acetate emulsion, and poly (vinyl alcohol) in the later stages of the MUF resin reaction to prevent excessive proximity of triazine rings. Some studies have also introduced flexible chain modifiers or functional group shielding modifiers to reduce cross-linking points on MUF [17,21,22,23,24]. Etherification toughening modification has garnered widespread attention due to its significant effects. Etherifying agents, typically alcohols such as methanol, ethanol, and butanol, can be used individually or in combination to improve MUF or melamine-formaldehyde (MF) resin properties [25,26]. Although etherified MUF resins have shown excellent flexibility and weather resistance in coatings, most are unsuitable for adhesives.

This research endeavors to enhance the toughness of MUF resin through the incorporation of poly (vinyl alcohol) (PVA). Its innovative aspects are twofold: Firstly, by integrating PVA, which features a long-chain flexible structure, the distance between triazine rings is increased, cross-linking density is reduced, and the flexibility of the MUF resin is bolstered at the molecular level. Secondly, a cold-setting MUF resin tailored for glued wood structures has been developed. This approach is anticipated to markedly boost the resin’s toughness and impact resistance, while preserving its superior bonding capabilities in glued wood production, thereby charting a novel course for the development and application of adhesives in the glued wood sector.

## 2. Materials and Methods

### 2.1. Materials

Melamine (99.8% purity), urea (99.0% purity), and poly (vinyl alcohol) (PVA) with (97.0% purity), all of analytical purity, were purchased from Sinopharm Chemical Reagent Co., Ltd., Shanghai, China. Formaldehyde (37% concentration) and sodium hydroxide (96.0% purity), both of analytical purity, were sourced from Chengdu Jinshan Chemical Reagents Co., Ltd., Chengdu, China; formic acid (88% concentration), of analytical purity, was sourced from Tianjin Fengshen Chemical Reagents Technology Co., Ltd., Tianjin, China. Rubberwood panels with a density of 0.65 g/cm^3^ and a moisture content of 10% were procured from Jingshan Wood Industry Department in Baoshan District, Shanghai, China.

### 2.2. Preparation of Melamine–Urea–Formaldehyde Resin

In a three-necked round-bottom flask fitted with a stirrer, thermometer, and condenser, formaldehyde was introduced into a 50 °C water bath. After adjusting the pH to 9.0, the first portions of urea and melamine were added, and the temperature was raised to 90 °C. The pH was then lowered to 5.0, and PVA was incorporated, with the reaction proceeding at 90 °C for 60 min. Subsequently, the pH was raised to 8.7–8.9, and the second portion of melamine was added, extending the reaction for 100 min. Finally, the pH was adjusted to 9.0. When the temperature decreased to 45 °C, the second portion of urea was added. After maintaining the temperature for 10 min, the pH was adjusted to 8.0 to complete the reaction, yielding the blank MUF resin (n(F): n(M + U) = 1.9:1). A series of resins were prepared by adjusting the PVA addition (0–10% of the total urea amount in 2% increments). The solid content, viscosity, pH value, and free formaldehyde content of these resins were measured according to GB/T 14074-2017 [27].

### 2.3. Preparation of Glued Wood and Shear Strength Testing

Glued wood samples were prepared in accordance with GB/T 26899-2011 [28] using a cold pressing technique. Par Rubber Wood sapwood specimens (30 mm × 25 mm × 10 mm) were coated with adhesive and allowed to stand at room temperature for 20 min before being pressed in a planar vulcanizing machine for curing. A WDS-50KN mechanical testing machine (Qingdao, China) was used to determine the shear strength at a loading rate of 2 mm/s. The process parameters included a pressure of 3.5 MPa, a press time of 2 h, an adhesive application rate of 300 g/m^2^, manual application, and a glue bonding area of 25 mm × 25 mm.

### 2.4. Water Resistance Test of Glued Wood

Rubberwood specimens measuring 75 mm × 50 mm × 10 mm were processed into glued wood using the previously described method. To assess water resistance in line with GB/T 26899-2011, the glued wood samples were subjected to immersion tests: 24 h in room-temperature water and 2 h in boiling water, followed by a peel test.

### 2.5. Tensile Property Test

**Figure 1:**
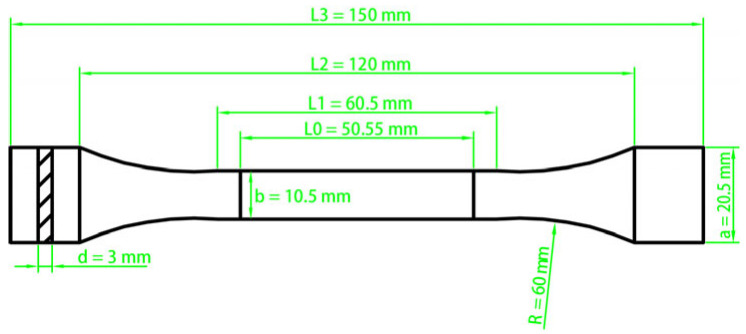
Dumbbell-shaped Model and size for tensile property test.

In accordance with GB/T 6344-2008 [29], the resin was molded into a “dumbbell-shaped” specimen and tested for tensile properties as seen in Figure 1. The elongation was determined by measuring the difference between the gauge length at break and the original gauge length during the test.

### 2.6. Characterization Analysis

X-ray Diffraction (XRD) tests were conducted using a TTR XRD (Tokyo, Japan) with a Cu target (λ = 0.154060 nm), scanning from 5–80° with a step size of 0.02° and a rate of 5°/min, at a tube current of 120 mA and voltage of 40 kV. Differential Scanning Calorimetry (DSC) analysis was performed using a DSC 204 F1 calorimeter from Netzsch, Rodgau, Germany, with a temperature range of 30–170 °C, a heating rate of 10 °C/min, nitrogen protection, and a sample mass of 5–8 mg. The fracture surface of the cured MUF resin layer was observed using a Hitachi S-3400N scanning electron microscope (SEM) from Tokyo, Japan, with gold coating and an acceleration voltage of 12.5 kV. For NMR analysis, a 100-μL sample of MUF resin and 300 μL of DMSO-d_6_ were mixed in an NMR tube and tested using a Bruker Avance high-resolution NMR spectrometer from Fallanden, Switzerland, with parameters including pulse sequence zgig, internal standard DMSO-d_6_, 500–800 scans, and a spectral width of 39,062.5 Hz.

### 2.7. Statistical Analysis

The data were processed using Excel 2021 and Origin 2024 software, and the significance of differences was judged via the one-way analysis of variance (ANOVA) (*p* < 0.05), the error bars represent the standard deviation.

## 3. Results and Discussion

### 3.1. Analysis of the Tensile Properties of MUF Resins

**Figure 2:**
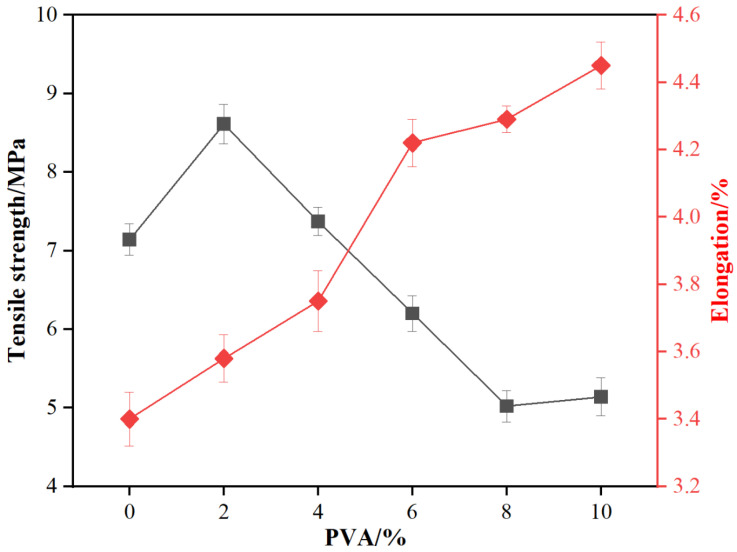
The tensile properties of MUF resins.

The PVA molecule, rich in hydroxyl groups (-OH), can form hydrogen bonds with amino groups (-NH_2_) and hydroxymethyl groups (-CH_2_OH) in MUF resin. These hydrogen bonds strengthen intermolecular interactions, thus boosting resin tensile strength. As shown in Figure 2, with increasing PVA addition, the tensile strength of MUF resin initially increases and then decreases. At a 2% PVA addition, the tensile strength reaches a maximum of 8.61 MPa, 20.6% higher than the blank resin. This is likely due to the good compatibility between PVA and MUF resin, which strengthens intermolecular interactions and raises tensile strength. However, as PVA addition continues to increase, tensile strength gradually decreases. Excessive PVA leads to phase separation or structural defects within the resin system, weakening overall tensile strength.

Regarding elongation, as PVA addition increases, the tensile elongation of MUF resin gradually rises. It increases from 3.40% of the blank resin to 4.45% at a 10% PVA addition, a total increase of 30.9%. This is mainly because the PVA molecular chain has certain flexibility and deformability, which allows better stress distribution during tensile deformation, thereby increasing tensile elongation. Additionally, PVA enables more even stress distribution in the resin system when subjected to external force, reducing stress concentration points and further increasing tensile elongation.

Toughness, which reflects an adhesive’s capacity to absorb energy and deform without brittle fracture under external force, is vital for bonding performance. It significantly enhances bonding strength, impact resistance, durability, processability, and adaptability to complex application environments. From the above results, it is evident that PVA addition significantly impacts the toughness of MUF resin. At a low addition (2%), the resin’s tensile strength is highest, but tensile elongation is relatively low, with toughness mainly reflected in high tensile strength. As PVA addition increases, tensile elongation gradually rises while tensile strength decreases, reaching a balance between tensile strength and deformation ability. At high additions (8% and 10%), tensile strength significantly decreases, but tensile elongation is high, with toughness mainly reflected in large deformation ability, better adapting to complex application environments.

### 3.2. Analysis of Bonding Performances

As shown in Table 1, with increasing PVA addition, the viscosity of MUF resin initially rises significantly and then gradually declines. At a 2% PVA addition, the resin viscosity reaches 1161.3 mPa·s, 24.5 times higher than the blank resin, whereas at a 10% PVA addition, the viscosity is 391.3 mPa·s, still higher than the blank resin. This may be attributed to the added PVA molecular chains, which increase intermolecular forces and entanglements in the resin system, thereby boosting viscosity. However, as PVA addition further increases, phase separation or structural defects within the resin system gradually emerge, resulting in a decrease in viscosity.

**Figure 3:**
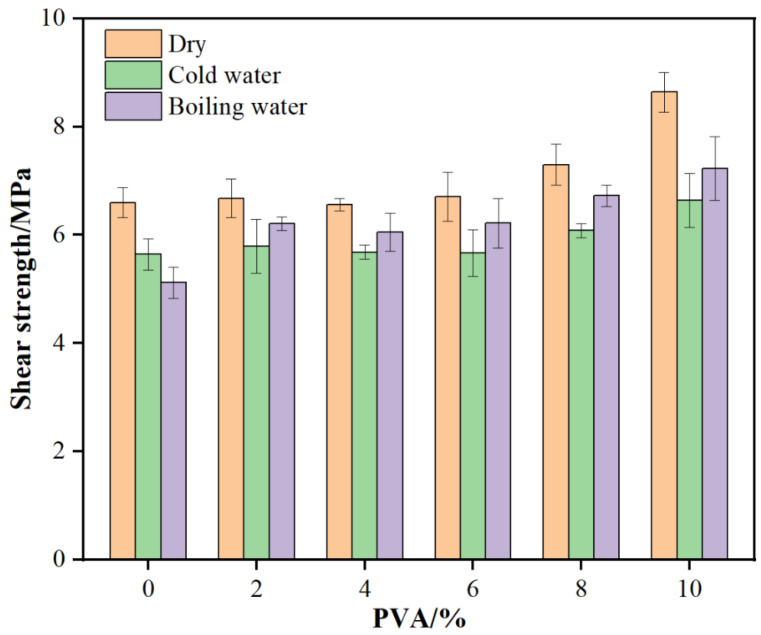
Bonding strength of glued wood with MUF resins.

From Figure 3, it can be seen that with increasing PVA addition, the dry gluing strength of glued wood initially increases and then stabilizes. At a 10% PVA addition, the dry gluing strength reaches a maximum of 8.64 MPa, 30.9% higher than the blank resin. This indicates that PVA addition significantly enhances the dry gluing strength of MUF resin, with the effect being more pronounced at higher additions. This may be due to the good compatibility and network structure formed between PVA and MUF resin, which strengthens the resin’s adhesion and internal cohesion with wood.

As PVA addition increases, the 24 h cold water gluing strength also initially rises and then stabilizes. At a 10% PVA addition, the 24 h cold water gluing strength reaches 6.64 MPa, 17.7% higher than the blank resin. This shows that PVA addition significantly improves MUF resin’s cold water gluing strength, enhancing its water resistance. This may be due to the flexibility and film-forming property of PVA molecules, enabling the resin to better maintain its structure and performance during cold water immersion. With increasing PVA addition, the 2 h boiling water gluing strength shows a significant upward trend. At a 10% PVA addition, the 2 h boiling water gluing strength reaches 7.23 MPa, 41.2% higher than the blank resin. This indicates that PVA addition significantly boosts MUF resin’s boiling water gluing strength, greatly improving its water resistance. This may be due to the interaction between PVA and MUF resin, which stabilizes the resin structure in high-temperature water environments and better resists water molecule erosion.

From the above results, it is clear that PVA addition significantly impacts the gluing performance of MUF resin. As PVA addition increases, MUF resin viscosity initially rises and then falls, but remains higher than the blank resin, which helps improve the resin’s coating performance and adhesion. Meanwhile, PVA addition significantly enhances the dry gluing strength, cold water gluing strength, and boiling water gluing strength of MUF resin, with the most noticeable effect at higher additions (10%). This suggests that PVA addition can effectively improve MUF resin’s water resistance and internal cohesion strength, making it better suited for wood gluing and other application requirements.

### 3.3. Analysis of Water Resistance

**Figure 4:**
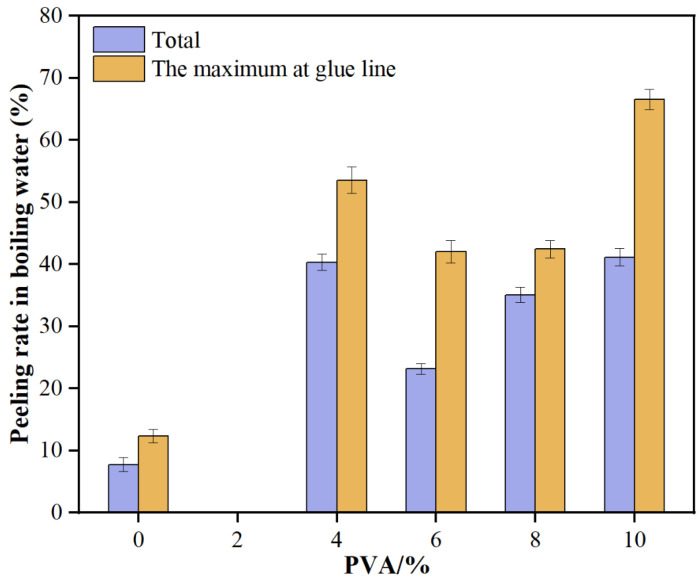
Water resistance of glued wood with MUF resins.

As illustrated in Figure 4, the total delamination rate of glued wood initially decreases and then increases with the rising amount of PVA added. Specifically, when 2% PVA is added, the total delamination rate drops to 0%, significantly lower than that of the blank resin. However, with 10% PVA, the rate surges to 41.13%. Similarly, the maximum delamination rate of a single layer follows the same trend, being 0% at 2% PVA and rising to 66.55% at 10% PVA. These results indicate that low levels of PVA enhance the water resistance of MUF resin, but higher amounts lead to a decline in this property.

From the above results, it can be seen that the addition of PVA has a significant improvement effect on the water resistance of MUF resin, but there is an optimal addition amount range. At a low addition amount of 2%, PVA can significantly reduce the total delamination rate and the maximum delamination rate of a single layer of glued wood, indicating that PVA forms a good compatibility and network structure with MUF resin, enhancing the water resistance of the resin. However, as the addition amount of PVA increases, the phase separation or structural defects within the resin system gradually become apparent, leading to a decrease in water resistance. This may be due to the excessive PVA causing uneven structures within the resin system, making stress more likely to concentrate under the erosion of water molecules, thereby increasing the delamination rate.

### 3.4. Analysis of DSC

**Figure 5:**
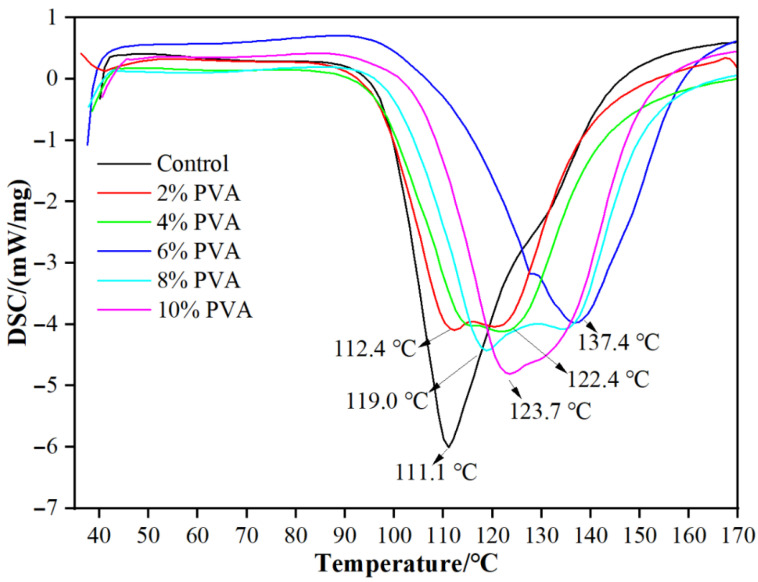
DSC curves of MUF resins.

From Figure 5, it can be seen that the curing temperature of the blank MUF resin is 111.1 °C. When the PVA addition amount is 2%, the curing temperature slightly increases (112.4 °C). This might be due to the formation of good compatibility between PVA and MUF resin, which enhances the interaction between molecules, resulting in a slight increase in the thermal stability of the resin system. The addition of PVA may have promoted the cross-linking reaction of MUF resin, thereby increasing the curing temperature. When the PVA addition amount is 4%, the curing temperature significantly increases to 122.4 °C; when the PVA addition amount is 6%, the curing temperature further increases to 137.4 °C. This indicates that the addition of PVA significantly increases the curing temperature of MUF resin. This significant increase might be due to the addition of PVA molecular chains, which increases the intermolecular forces and cross-linking density of the resin system. The flexibility and film-forming property of PVA molecules might have hindered the curing reaction of MUF resin to some extent, causing the curing temperature to rise. When the PVA addition amount is 8%, the curing temperature drops to 119.0 °C; when the PVA addition amount is 10%, the curing temperature is 123.7 °C. This indicates that as the PVA addition amount increases further, the curing temperature decreases. This might be due to the formation of phase separation or structural defects within the resin system caused by excessive PVA, weakening the overall thermal stability of the resin system. Additionally, excessive PVA might dilute the cross-linking reaction of MUF resin to some extent, thereby lowering the curing temperature. In summary, the addition of PVA increases the curing temperature of MUF resin.

### 3.5. Analysis of NMR

**Figure 6:**
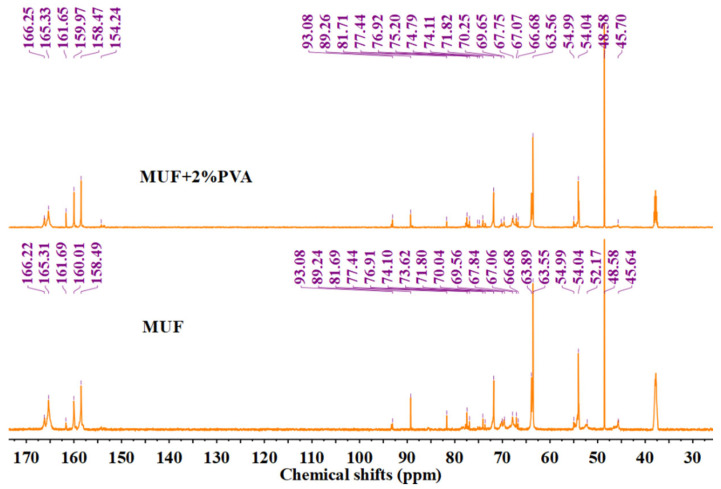
^13^C-NMR curves of MUF resins.

The carbon–oxygen double bond in urea and the triazine ring in melamine will not break during the reaction process. Their chemical shifts are 157–162 ppm and 166–167 ppm, respectively. The methoxy carbon belongs to methanol and does not participate in the reaction. The unreacted formaldehyde exists in the form of methanediol (with a chemical shift of 83 ppm), and the integral of all absorption peaks is calculated based on this peak. Then, the sum of the integral areas of all methylene carbons is calculated, and the ratio of the integral values of each type of chemical bond to the total integral value of methylene carbons is calculated as the percentage of each type of methylene carbon [30,31,32,33]. The test results are presented in Figure 6 and Table 2. The hydroxymethyl group, a product of an addition reaction, is essential for resin molecular chain extension and cross-linking. After condensation, bridge and ether bonds form, consuming the hydroxymethyl groups. A higher proportion of methylene bridge and ether bonds suggests greater resin polymerization, which correlates with increased resin strength.

The chemical shifts for different groups are as follows: the hydroxymethyl group appears at 63–65 ppm, methylene bridge bonds at 54–56 ppm and 46–48 ppm, and methylene ether bonds primarily at 67–70 ppm. Additionally, the methylene ether bonds resulting from the copolymerization of melamine and urea are observed at 74–75 ppm and 77–78 ppm. In the unmodified MUF resin, the content of hydroxymethyl is 45.35%, the methylene bridge bonds are 17.08%, the methylene ether bonds are 15.53%, and the methylene ether bonds produced by the copolymerization reaction of melamine and urea are 10.76%. In MUF/PVA, the content of hydroxymethyl is 41.37%, the methylene bridge bonds are 14.01%, the methylene ether bonds are 23.55%, and the methylene ether bonds produced by the copolymerization reaction of melamine and urea are 11.22%. The content of methylene bridge and ether bonds in MUF and MUF/PVA resins is 43.37% (17.08% + 15.53% + 10.76%) and 48.78% (14.01% + 23.55% + 11.22%), respectively. Compared with MUF, the MUF/PVA system has more hydroxymethyl participating in the polymerization reaction, a larger total amount of methylene bridge ether bonds, and a higher degree of polymerization, thus showing better mechanical properties. However, the decrease in the content of methylene bridge bonds and the increase in the content of ether bonds indicate that the introduction of PVA affects the competitive reaction between bridge bonds and ether bonds, inhibiting the formation of bridge bonds.

The chemical shifts of PVA should be 67.6–68.2 ppm and 40–50 ppm. If PVA undergoes a condensation reaction with formaldehyde, new peaks will be generated at the chemical shifts of 86.0 (the external diastereoisomer vibration of the methylene carbon) and 92.4 (the internal diastereoisomer vibration of the methylene carbon), and the reaction activity of the internal diastereoisomer is much greater than that of the external diastereoisomer, making it easier to detect. By comparing Figure 2 and Figure 3, it can be seen that the characteristic peaks of PVA are at 45.1–46.3 ppm and 67.2 ppm. No new peaks are generated near the chemical shifts of 86.0 and 92.4 ppm, so it cannot be determined whether PVA and formaldehyde have undergone the PVA condensation with formaldehyde reaction.

### 3.6. Analysis of XRD

**Figure 7:**
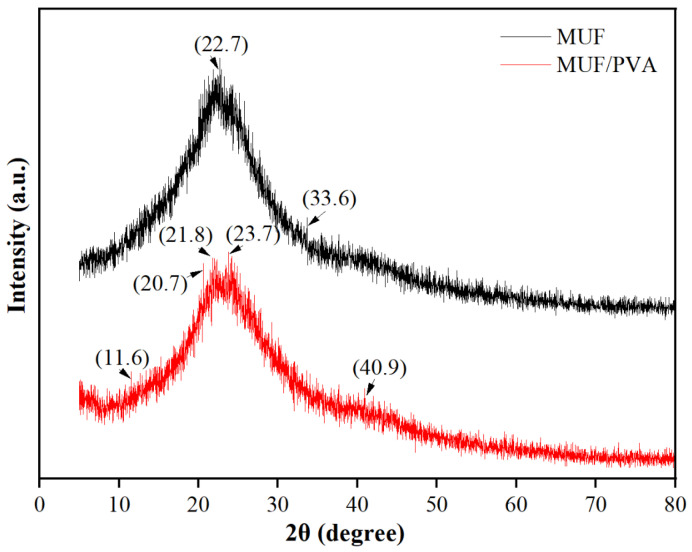
XRD curves of MUF resins.

As shown in Figure 7, the MUF resin exhibits distinct diffraction peaks, indicating that it has a certain degree of crystallinity. The main diffraction peaks are observed at 2θ values of approximately 22.7° and 33.6°, which reveal the specific crystal structure of the MUF resin. Compared with pure MUF, the diffraction peaks of MUF/PVA are slightly reduced, and new diffraction peaks appear at 2θ values of approximately 11.6°, 20.7°, and 40.9°. The decrease in crystallinity may indicate an increase in the amorphous regions within the material. The amorphous regions have higher molecular chain activity and less long-range order, which enables the material to deform plastically more easily when subjected to external forces, thereby absorbing more energy. The enhanced energy absorption capability boosts the material’s toughness, which is its ability to deform without brittle fracture under external forces. The new diffraction peaks suggest that PVA addition may have induced the formation of new crystal phases. These new crystal phases may have higher toughness or better stress transfer ability, thereby affecting the overall toughness of the composite material. Additionally, hydrogen bond interactions between PVA molecular chains and MUF resin molecular chains may have formed, which not only enhance the stress transfer efficiency within the material but also promote cooperative deformation between molecular chains, further improving toughness.

NMR analysis shows that PVA addition enhances the polycondensation of MUF resin, boosting methylene bridge and ether bond content, which improves resin mechanical properties. Additionally, PVA inhibits methylene bridge formation while increasing ether bond content, possibly explaining the reduced crystallinity and new crystal phases seen in XRD results. The increase in ether bonds may make the interactions between molecular chains more flexible, thereby increasing the proportion of amorphous regions and promoting the formation of new crystal phases. These structural changes work together to enable MUF/PVA to maintain a certain strength while also improving toughness.

### 3.7. Analysis of SEM

**Figure 8:**
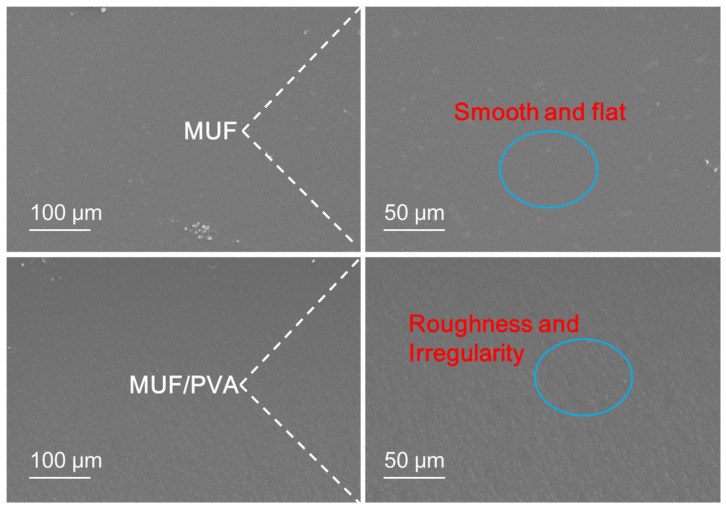
SEM of the cross section of MUF resins.

From the observations of the scanning electron microscope in Figure 8, the fracture surface of pure MUF resin presents a smooth and flat appearance, which is a typical sign of brittle fracture [34,35]. In this fracture mode, the stress concentration within the material will rapidly trigger the expansion of cracks, and during the crack expansion process, almost no plastic deformation occurs, resulting in the material being prone to sudden fracture when subjected to impact. In contrast, the fracture surface of MUF resin modified by PVA shows obvious “grooves” and rough and irregular surface textures. This unique surface feature reveals that the modified resin undergoes ductile fracture during the fracture process. Ductile fracture is a more favorable fracture mode, involving plastic deformation within the material and effective energy absorption. During ductile fracture, the material can disperse and absorb more energy through plastic deformation, thereby significantly enhancing its impact resistance. This fracture mode enables the material to better withstand stress without undergoing brittle fractures when subjected to external forces, thereby improving the durability and reliability of the material.

The introduction of PVA may promote the occurrence of ductile fracture through multiple mechanisms. Firstly, hydrogen bond interactions between PVA molecular chains and MUF resin molecular chains are formed, which enhance the intermolecular bonding force and enable the material to more effectively transfer stress when subjected to external forces. Secondly, the addition of PVA increases the proportion of amorphous regions in the material, which have higher molecular chain activity and can better absorb and disperse energy. Additionally, the addition of PVA may promote the formation of new crystal phases, which have higher toughness and better stress transmission capabilities, further enhancing the overall performance of the material.

From the previous NMR analysis results, the addition of PVA promotes the polycondensation reaction of MUF resin, increasing the content of methylene bridge bonds and ether bonds, thereby improving the mechanical properties of the resin. At the same time, the addition of PVA inhibits the formation of methylene bridge bonds and increases the content of ether bonds, which may be related to the ductile fracture characteristics observed in the SEM results. The increase in ether bonds may make the molecular chains interact more flexibly, thereby increasing the proportion of amorphous regions and promoting the occurrence of ductile fracture. These structural changes work together to enable the MUF/PVA composite material to more effectively absorb energy and undergo plastic deformation when subjected to external forces. Through PVA modification, not only is the material’s toughness improved, but it can also more effectively disperse stress when subjected to impact or load, reducing the formation and expansion of cracks, thereby significantly enhancing the durability and reliability of the material. These structural and fractural changes are crucial for the application performance of the material, especially in those fields that have strict requirements for toughness and impact resistance.

## 4. Conclusions

This study performed a series of performance tests and structural analyses on MUF resins modified with varying amounts of poly (vinyl alcohol) (PVA), and arrived at the following conclusions:

1. The addition of PVA significantly enhanced the tensile strength and elongation of MUF resins. When the PVA addition amount was 2%, the tensile strength reached its maximum of 8.61 MPa, which was 20.6% higher than that of the unmodified MUF resin (7.14 MPa). The elongation at break increased from 3.40% (unmodified) to 4.45% (10% PVA addition), representing a 30.9% improvement.
2. The addition of PVA significantly increased the dry, cold water, and boiling water bonding strengths of MUF resins, and the effect was most pronounced at a 10% addition level. The dry-state bonding strength reached 8.64 MPa, which was 30.9% higher than that of the unmodified MUF resin (6.60 MPa); the 24 h cold bonding strength reached 6.64 MPa, which was 17.7% higher than that of the unmodified MUF resin (5.64 MPa); the 2 h boiling water bonding strength reached 7.23 MPa, which was 41.2% higher than that of the unmodified MUF resin (5.12 MPa).
3. The DSC analysis results indicated that the addition of PVA promoted the cross-linking reaction of MUF resins and increased their curing temperature. NMR analysis revealed the changes in chemical bonds in the PVA-modified resins, indicating an increase in the degree of polymerization, thereby enhancing the mechanical properties of the resins. XRD and SEM analyses further confirmed the effects of PVA modification on the crystal structure and microscopic morphology of MUF resins, with the modified resins showing higher toughness fracture characteristics.

## Figures and Tables

**Table 1:** The solid content and viscosity of MUF resins.

PVA/%	Solid Content/%	Viscosity/(mPa·s)
0	49.67 (0.23)	46.19 (0.13)
2	51.17 (0.62)	1161.30 (0.08)
4	51.99 (0.22)	181.40 (0.11)
6	51.95 (0.05)	247.87 (0.17)
8	51.46 (0.38)	215.17 (0.31)
10	52.80 (0.19)	391.30 (0.36)

**Table 2:** Percentage Values for Various Methylenic Carbons of MUF Resins.

Structures	Chemical Shifts/ppm	MUF	MUF/PVA
-NH-CH_2_-NH-	46–48	9.40	9.29
-N(CH_2_-)-CH_2_-NH-	54–56	7.68	4.72
CH_3_OH/-CH_2_OCH_3_	49–50		
M-NH-CH_2_OH/U-NH-CH_2_OH	63–65	45.35	41.37
-NHCH_2_OCH_2_(NH-/-OH)	67–70	15.53	23.55
-NHCH_2_OCH_3_	72–73	9.08	8.06
M-N(CH_2_-)-CH_2_-O-CH_2_-N(CH_2_-)-U	74–75	10.76	11.22
M-NH-CH_2_-O-CH_2_-N(CH_2_-)-U	77–78		
HOCH_2_OH	82.4–82.5	2.20	1.79
HOCH_2_OCH_2_OH	86.2–86.3		

## Data Availability

The original contributions presented in this study are included in the article. Further inquiries can be directed to the corresponding authors.
